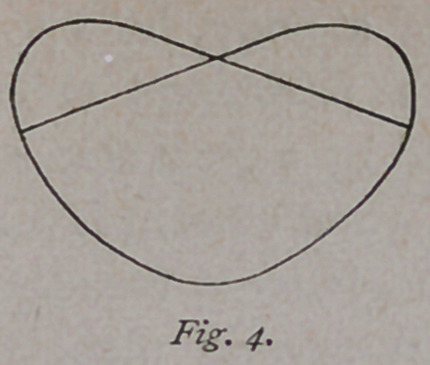# The Rectification of Face Presentations

**Published:** 1889-12

**Authors:** Rollin L. Banta

**Affiliations:** Buffalo, N. Y.; Vice-President American Association of Obstetricians and Gynecologists; Consulting Obstetrician to the Sisters of Charity and Maternity Hospitals; 358 South Division Street


### Buffalo Medical and Surgical Journal

Vol. XXIX. DECEMBER, 1889. No. 5.

### Original Communications.

### THE RECTIFICATION OF FACE PRESENTATIONS.[1]

By ROLLIN L. BANTA, M. D., Buffalo, N. Y.

Vice-President American Association of Obstetricians and Gynecologists; Consulting Obstetrician to the Sisters of Charity and Maternity Hospitals.

Because of the uncertainties of the prognosis in face presentations, many manœuvres have been proposed for the conversion of the latter into normal presentations. Though occasionally successful, they have been discountenanced by most obstetric writers, because experience has shown the results to be by no means commensurate with the dangers incurred.

This quotation, taken from one of our latest obstetrical writers, seems to be, as far as my observation goes, the sentiment of most of the eminent obstetricians of the present day. All agree that face births are abnormal births, and although this form of labor may be a very simple one, as a rule, it is attended with more danger to the mother, and is manifoldly more dangerous to the child. Also, it seems to be almost the unanimous opinion that the various methods proposed by such men as Meigs, Baudelocque, Hodge, and lately by Shatz, either on account of the difficulties or the dangers which occur in putting them into practice, have not succeeded in fulfilling the hopes entertained by their originators; and, all things being considered, it is better to trust to nature than to any manipulation, although, in a great many cases, nature makes pretty bad work of it, and often is utterly unable to accomplish her object, without the aid of art. My own experience is so at variance with these teachings that it is to be hoped that it will not seem presumptuous in my claiming that face presentations can be rectified by internal manipulation; and that an abnormal and tedious labor can be easily and safely changed into a normal and simple one, thereby saving much suffering to the mother, and danger to the child.

If, at times, a beaten path is gone over on the plan presented in this paper, it is hoped enough of the new is added, and the whole so

1. Read at the annual meeting of the American Association of Obstetricians and Gynecologists in Cincinnati, O., September 17, 1889.

simplified and methodized, that a claim can be made of touching at least the boundary line of originality.

For convenience of description, and for the reason that the writer has found it to answer his purpose so admirably—and, moreover, because it is believed to be right—a brief sketch of the anatomy of the female pelvis, as taught by the late Dr. Henry C. Landis, is here given.

If the sacral and iliac wings of the female pelvis be removed, the inlet presents an appearance as shown in Fig. 1.[1] For all practical purposes, the outlet has a shape as shown in Fig. 2. If these two figures be combined, and drawn lines to indicate the pelvic walls, we have an outline as shown in Fig. 3. If, now, a card-board be cut out having the shape of a section of the middle circumference of the flexed head, it has the shape of an ellipse, as shown in Fig. 2, which also represents the outlet. If the card is now applied to the inlet, it is found that it completely coincides with it on one side, and, if reversed on the other side, the two outlines intersect each other.

*Fig. 1.*

It, therefore, requires but a slight stretch of the imagination to see that the inlet, instead of being an irregular oval, as is generally described, is, indeed, "beautifully regular in outline, and that the pelvis contains two canals, each of the same outline or calibre as the fetal head, and a little larger. These canals are partly divergent above, and entirely identical at the outlet," as shown in the theoretical diagram (Fig. 3). The one containing the right oblique diameter is called the right canal, the opposite one the left canal. The head, in labor, passes into one o these canals, and, as it descends into the pelvis, it is, of course, followed by the shoulders, also having an elliptical outline, which enter the other canal. Before the shoulders descend very far, the head is born, showing there is need of one canal only at the outlet.

*Fig. 2.*

*Fig. 3.*

One more glance at the pelvis, in order to call attention to certain spaces bounded in front by imaginary lines drawn from the base of each sacro-iliac arch, as shown in Fig. 4. These lines may be called the sacro-cotyloid diameters, and the spaces posterior to them, for want of a better name, the sacro-cotyloid spaces. They

1. Figures 1, 2 and 3 are taken from Dr. Henry C. Landis' "How to Use the Forceps."

are situated at the posterior part of the right and left oblique diameters, or, in other words, at the posterior part of the right and left canals. At the inlet they are quite conspicuous, and become smaller as the canals merge into one; so that a body occupying one of the canals, there is necessarily left at the posterior part of the other a free space, which disappears as the body descends into the pelvis. Special attention is called to this space, as it has an important bearing on what follows.

*Fig. 4.*

Face births are divided into anterior and posterior presentations. When the chin presents anteriorly, if the pelvis is large, the head small, or the neck long, there is usually no difficulty in delivery; but, if otherwise (and how any one can tell the size of the head or neck before delivery, has never been in my power to discover), then the birth becomes a more serious matter. A body having a diameter of about seven inches, must be forced through a tube, the longest diameter of which is a little over four inches. The characteristic appearance of the child's head after a face birth shows the great amount of moulding which must take place before delivery is accomplished. If, then, a certain number of anterior face presentations are so serious in their nature, any procedure which will safely rectify and make them normal will certainly be a boon to the mother, saying nothing of the avoidance of the dangers which threaten the child. Such a procedure it is now my intention to describe, and it has been put to a test often enough to leave, in my opinion, no doubt of its efficacy.

It is not questioned for a moment that quite a proportion of face presentations are easy births; but to tell just which ones to interfere with, is a question extremely difficult to decide; therefore, would it not always be better to rectify them as early as possible, especially as there is not the slightest doubt that those cases which nature can take care of are easily managed, while those of a more serious character must often depend upon artificial aid?

Before proceeding further, it will be well to state that the application of the method under consideration necessitates that the head is movable and not impacted in the pelvic cavity, and that the cervix is dilated, or sufficiently dilatable, for the introduction of the hand into the uterus.

In the first face presentation, L. M. A., the face is in the right canal, and the trachelo-bregmatic diameter of the face corresponds to the long diameter of the right canal, or to the right oblique diameter of the inlet (German nomenclature). Even at the inlet, the face entirely fills this canal. As the head descends, the anterio-posterior diameter of the neck, about three inches, is added to the depth of the cranium or to the suboccipito-bregmatic diameter of the head, about four inches in length. Now begins the process of a body, having a diameter of seven inches, being forced through a passage of about four inches, which, if left to nature, is accomplished by a great amount of force and moulding of the parts, and only then when certain favorable conditions, before mentioned, are present. A glance at the other canal shows that it is occupied by the bimalar or transverse plane of the face, but, on account of the smaller size of this diameter or plane, measuring a little over three inches across, there is left at the posterior part of this canal a free space, the left sacro-cotyloid space. It would seem, on first thought, if there were any internal manipulations to be made above the brim, it would be best to pass the right hand through this unoccupied space; but to reach the posterior part of the head, which will be seen in a moment is one of the main objects to be attained, it would be necessary to pass the hand from one side of the pelvis to the other, over or under the shoulders or body of the child, which for any practical purposes would be impossible.

In order, then, to manipulate the occiput or extended head which occupies the right sacro-cotyloid space, the left hand is passed into the vagina and uterus (if need be) on the right side of the pelvis wall back until it is stopped by the forehead or vertex. By now placing the fingers around the vertex, or, for that matter, any part that can conveniently be grasped, only remembering that the hand is on the right side and to the posterior, enough force can be used, and only the slightest amount of force is required, to rotate the head anteriorly about a quarter of a circle, when the chin will look posteriorly and the face will be in the left canal. At the same time, and, perhaps, just before the attempt is made to rotate, the head is pushed up sufficiently so that the chin will be well above the brim. This is a very important point to remember if the head is large or the pelvis small, and even if that is not the case, it may save considerable trouble in the after-manipulations. The face is now in the L. M. P. position, which is considered one of the most difficult for delivery. There is also at the posterior part of the right canal a free space, the right sacro-cotyloid space, through which the hand is passed and placed well over the occiput. The next step is to bring about flexion, which is not always easily accomplished on account of the cramped position of the internal hand.

Every obstetrician is well aware of the fact that occasionally it is almost impossible to keep the hand within the contracting uterus for any length of time, and if there is work to do it must be done quickly, or the hand withdrawn for a rest. This difficulty can usually be overcome by placing the free, or right hand, over the abdomen of the mother and over the left internal hand, when flexion, or, for that matter, extension, can be made at will. After flexion the presentation is R. O. A., or the old second position. The right hand is an important factor in this manœuvre, for it not only aids the left hand in flexing the head, but it also helps to push it well down into the pelvic cavity. If flexion does not readily occur, it is because the chin has not been forced high enough above the brim and it is stopped at some point of the pelvic walls. If there is any fear that extension will again take place, and that is liable to happen only when there has been a failure to force the flexed head well down into the pelvis, either the hands should be kept in place for a few pains, or the forceps applied and enough traction made to prevent that danger. A knowledge of the manner of proceeding in the position just described—the first face position—is the key to the other three, consequently only a few remarks need be made concerning each of them.

In the R. M. A. position the face is in the left canal, and now, for the same reason, the right hand is used instead of the left. The occiput is brought to the front in the same manner, and having passed the hand through the left sacro-cotyloid space, the head, with the aid of the left or free hand, is flexed and pushed down into the pelvis, which changes the position into the L. O. A., or old first position.

In mento-anterior positions when the child's back looks posteriorly, forced anterior rotation of the head may twist the child's neck disagreeably and perhaps dangerously; but in the cases which have come under my observation, rotation of the body has always accompanied rotation of the head. The judgment of the past and present is adverse to any interference with anterior face presentations, but the certainty that experience gives one of doing a thing safely and well has robbed for me that variety of births of any anxiety when called to attend them. With our knowledge of modern antiseptics, the introduction of a clean hand into the uterus, through a clean vulva and vagina, is entirely devoid of danger as far as carrying any septic material is concerned. Adding to this a certain amount of skill, which every practitioner is supposed to possess, there is scarcely any doubt of the successful application of the rules just described.

Regarding mento-posterior presentations, my method of rectification is so similar to that described by others, that if it were not for some change in the technique of the manipulations, it would hardly be worthy of repetition. Sometimes a few slight additions to an operation will make easy what was before difficult. At any rate, four posterior presentations, one of them a brow, which may be considered a variety of face birth, have been rectified by the following plan with the happiest results, only one causing any considerable trouble:

Nature's method of delivery, when the chin looks to the posterior, is anterior rotation, and if that does not take place, Nature is unable to finish her task, unless under very unusual circumstances; because the "forehead remains stationary at the first part of the brim, while the base of the skull and the upper part of the chest attempt to advance under the sacro-iliac arch, which is impracticable." It is for this reason that it has been proposed lately to bring about anterior rotation by manual interference and the subsequent application of the forceps, which is, indeed, a very good plan if it were not possible to substitute a better one. In the R. M. P. position, the face being in the right canal, all that is necessary to do is to produce flexion, keeping the long diameter of the face and head always in the same canal. In fact, it is a repetition of the same steps as in the L. M. A. position after rotation has been effected. In the same way, the right hand is passed into the right sacro-cotyloid space, when, with the aid of the left hand, the head is flexed and pushed down into the pelvic cavity, and into the R. O. A. position. It is well not to forget that one of the principal points essential to the success of the manœuvre, before an attempt has been made to bring about flexion, is to see that the chin is forced well above the brim. In the L. M. P. position, as the face is in the left canal, the left hand is used instead of the right, and the remaining steps are exactly the same as those just described.

Like almost every other obstetrical operation where it is necessary to make internal manipulations, it is best to bring the patient to the edge of the bed as in the forceps position. In the majority of cases it will be found impossible to do anything without administering an anesthetic, although in three of the five anterior mento-presentations under my care it was not employed, and the whole manœuvre was performed in considerable less time than it took to explain the trouble and get the consent of the patient.

358 South Division Street.

---

Ergot in labor is advised by Schatz (*Deutsche Med. Zeitung*) in the form only of fresh fluid extract, whenever the pains are feeble. It produces normal pains which are not increased in intensity, but in number, if used in ten or twelve drop doses—more frequently than is now the custom. [We think ergot in any form is not needed in labor.—Ed.]

## Figures and Tables

**Fig. 1. f1:**
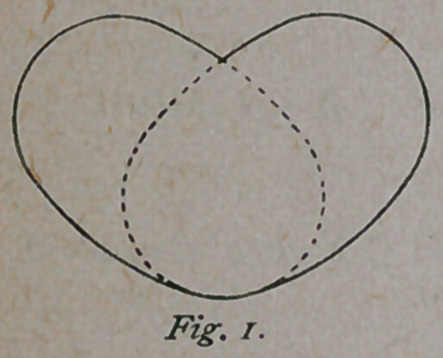


**Fig. 2. f2:**
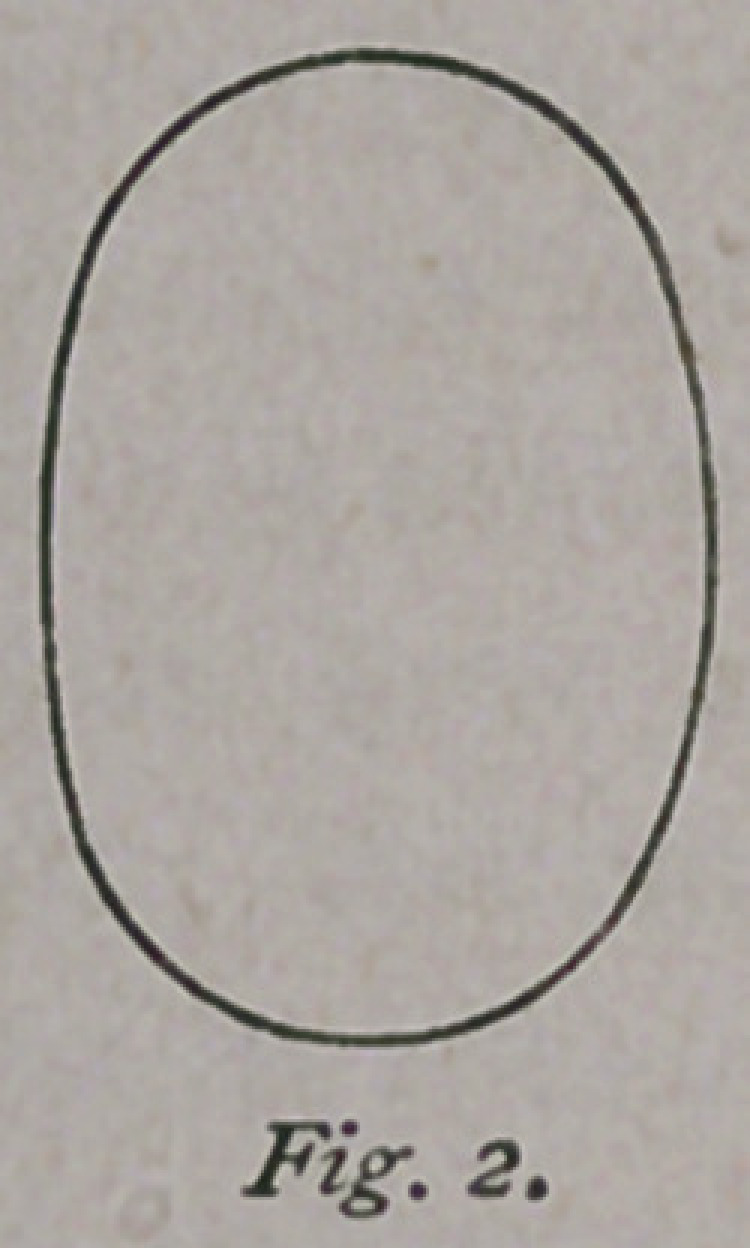


**Fig. 3. f3:**
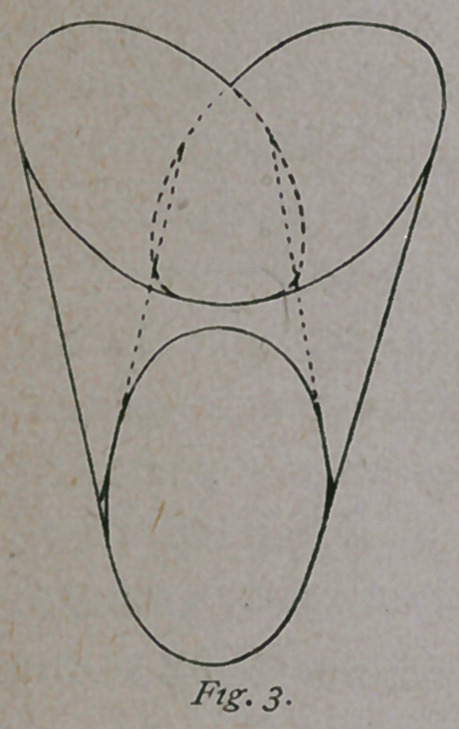


**Fig. 4. f4:**